# Non‐Native Herpetofauna Continue to Proliferate in the World's Most Invaded Herpetofauna Community

**DOI:** 10.1002/ece3.71556

**Published:** 2025-06-13

**Authors:** Stephanie L. Clements, Frederick M. Ackerman, Isabella M. Olensky, Elizabeth C. White, Millie E. Rogers, Christopher A. Searcy

**Affiliations:** ^1^ Department of Biology University of Miami Coral Gables Florida USA; ^2^ Tropical Audubon Society Miami Florida USA; ^3^ Tulane University School of Medicine New Orleans Louisiana USA; ^4^ Nicholas School of the Environment Duke University Durham North Carolina USA

**Keywords:** amphibian, conservation, Florida, invasive species, reptile, urban ecology

## Abstract

The spread of non‐native species continues to increase around the globe, highlighting the importance of understanding both the dynamics of the invaded communities in which non‐natives comprise a high percentage of the total fauna and the factors that may influence non‐native establishment and spread. As the global hotspot for non‐native reptiles and amphibians, South Florida's herpetofaunal community provides a unique opportunity to study native/non‐native community dynamics. In addition, despite high levels of development and habitat loss, South Florida has a network of protected natural areas, allowing insight into the impacts of natural vs. anthropogenic areas on native and non‐native richness and abundance. Surveys conducted in Miami‐Dade County in 2017 demonstrated that non‐native species already dominated both natural and anthropogenic parks and provided a baseline to examine dynamic changes in the community. In 2022, we replicated the surveys from 2017 at the same 30 sites. We found that non‐native richness and abundance have increased significantly (19% and 33% increase in overall alpha diversity and abundance, respectively) in just 5 years' time. We found no correlation between these non‐native increases and decreases in either native species richness or abundance. Notably, non‐native species richness increased more rapidly at anthropogenic sites, with two rock‐loving species, *Agama picticauda* and *Leiocephalus carinatus* , standing out as the most rapidly spreading non‐native herpetofauna. Our findings demonstrate that there is continued expansion of non‐native herpetofaunal populations even in the highly invaded community of Miami‐Dade County and that protection of natural habitat may help slow the spread of non‐native species.

## Graphical abstract

South Florida has the most non‐native reptiles and amphibians of anywhere in the world, making it the most likely herpetofaunal community to have reached saturation, yet we found significant increases in both alpha diversity and abundance over the last 5 years. Non‐natives increased most rapidly at sites dominated by an anthropogenic habitat, indicating that protection of natural habitat may help slow the spread of non‐native species. Two rock‐loving lizards, *Agama picticauda* and *Leiocephalus carinatus* , stood out as the most rapidly spreading non‐native herpetofauna.

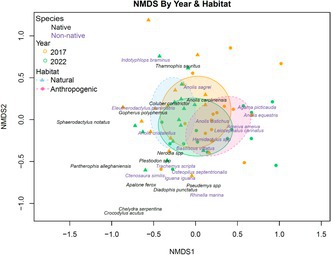

## 1 Introduction

Human population growth, increased urbanization, and other anthropogenic activities like global trade and ornamental landscaping have led to the spread of non‐native species on a global scale (Jeschke et al. 2014; Pyšek et al. 2020), while the establishment of non‐native species is made even more likely through climate change and habitat modification (Byers 2002; Perrings et al. 2005; D'Amore et al. 2010; Banks et al. 2015; Marques et al. 2020). As species spread to new areas, they can impact the balance of an ecosystem, outcompeting or predating native species (Honek et al. 2016; Haubrock et al. 2020; Bando et al. 2023). In addition to ecological damage, non‐native species also cause economic damage. Globally, the estimated economic cost of biological invasions was $423 billion in 2019 (Schwindt et al. 2023), and in the United States alone, an estimated $21 billion/year is lost due to invasive species (Fantle‐lepczyk et al. 2022). The worsening spread and increased abundance of non‐native species (Pyšek et al. 2020) underscores the importance of understanding the factors that influence non‐native spread, how non‐native species are changing ecological communities, and the impact they can have on native species (Seebens et al. 2017).

Non‐native species have been the second most common cause of species extinctions, after habitat destruction, since 1500 AD, and are the most significant threat to the extinctions of amphibians, reptiles, and mammals (Bellard et al. 2017). Within numerous systems, non‐native species have been documented as one of the chief reasons for declines in native species richness (Dorcas et al. 2012), shifts in native species composition (Honek et al. 2016), and community homogenization (Bando et al. 2023). Additionally, hybridization between native and non‐native individuals can result in the replacement of native populations with hybridized individuals (Kraus 2015; Wegener et al. 2019). Negative ecological impacts of non‐native species include predation on native species, the transmission of diseases, and increased resource competition (Rodda and Savidge 2007; Picco and Collins 2008; Cole and Harris 2011; Kraus 2015). The last of these is most likely to occur between species with similar ecological roles and, given phylogenetic signal in such traits, is most often observed between native/non‐native congeneric pairs (Gioria and Osborne 2014; Zwerschke et al. 2018). As non‐native species move into new communities, it is also possible that these communities could reach a saturation point at which either these non‐native species cannot continue to encroach, or native species are lost to make room for new non‐natives (Starzomski et al. 2008; Pinto‐Sánchez et al. 2014; Sax and Gaines 2008).

Habitat destruction, in addition to being the leading cause of biodiversity loss around the globe, facilitates the spread of non‐native species, leading to potential compounding effects (Macdougall and Turkington 2010; Marvier et al. 2004). Urbanization continually degrades and destroys native habitats by converting natural spaces to those with built structures and impervious surfaces (Marques et al. 2020). Non‐native species have been found to exist at higher abundances in altered systems, and their success is likely due to the environmental conditions caused by human modification (Cadotte et al. 2017; Howell et al. 2021). Non‐native species also often prefer disturbed habitats with hydrological alterations, fragmentation, and degradation of natural areas (D'Amore et al. 2010; Howell et al. 2021). At the same time, native species are negatively impacted by disturbed and degraded habitats, creating open niche space for non‐native species in these environments (Byers 2002; D'Amore et al. 2010). Therefore, disturbed areas are more likely to hold higher numbers of non‐native species, while also being detrimental for native species (Kraus 2015).

South Florida, a region experiencing significant habitat change, is recognized as the most highly invaded continental ecoregion in the world (Searcy et al. 2023). Numerous factors contribute to the large number of non‐native species in Florida, including the subtropical climate, high levels of natural disturbance, maritime traffic, and the thriving exotic species trade (Smith 2005; Krysko et al. 2009; Fujisaki et al. 2009; Fieldsend et al. 2021; Searcy et al. 2023). Additionally, climate change will likely influence which species (both native and non‐native) thrive in South Florida as the ecosystems are altered by temperature, precipitation, and drought regime changes (Nungesser et al. 2015; Stys et al. 2017; Dubos et al. 2023). The loss of natural habitat in South Florida due to land use change continually threatens native biodiversity and provides a foundation for non‐native species to flourish (Divya et al. 2021; Marques et al. 2020). Despite initiatives to increase the footprint of protected native habitat in Miami (Diamond and Heinen 2016; Alonso and Heinen 2011), urban development has increased 200% over the last 20 years (Divya et al. 2021).

Florida has the largest number of established non‐native amphibian and reptile species in the world (Capinha et al. 2017), many of which have been present in the state for several decades. The oldest introductions occurred ~150 years ago with the brown anole *Anolis sagrei* ) introduced in 1887 (Garman 1887) and the greenhouse frog *Eleutherodactylus planirostris* ) introduced in 1863 (Cope 1863). In 1958, only 12 non‐native herpetofauna species were recorded in Florida compared to the 63 recorded as of 2016, highlighting the dramatic increases in non‐natives in this region (Duellman and Schwartz 1958; Krysko et al. 2016). As such, South Florida's highly invaded herpetofaunal community provides a unique opportunity for tracking the spread of non‐native species as well as the impact that these species may have on the native community.

There are substantial records of non‐native species introductions in Florida, as well as of increases in the abundance of non‐native species (Krysko et al. 2009, 2011, 2016). However, there are limited studies that have investigated changes in local abundance and alpha diversity across defined time scales. Cassani et al. (2015) scratched the surface of investigating change over time by finding significant increases in the brown anole, *Anolis sagrei* , and concluding that herpetofaunal communities are changing rapidly in their study region of southwest Florida. However, this study was conducted in a large natural habitat parcel and not in urbanized areas (Cassani et al. 2015). As discussed above, non‐native species generally thrive in more disturbed areas, like the highly urbanized portions of Miami‐Dade County (Byers 2002; Clements et al. 2019; Marques et al. 2020). A survey completed in 2017 revealed that non‐native species dominated the herpetofaunal community in both anthropogenic and natural areas in Miami‐Dade County (Clements et al. 2019). This previous survey, spanning 30 sites, provides a baseline to allow for investigation of how the herpetofaunal community is changing over time, factors that influence that change, and whether non‐native species are having an impact on native species richness or abundance. To address these questions, we replicated the surveys from 2017 at the same sites and during the same time of year (Clements et al. 2019). The ability to re‐survey the same sites presents us with a unique opportunity to investigate the changes in the herpetofaunal community of South Florida, the global hotspot for non‐native species, over a span of just 5 years.

## 2 Methods

### 2.1 Site Selection

**FIGURE 1:**
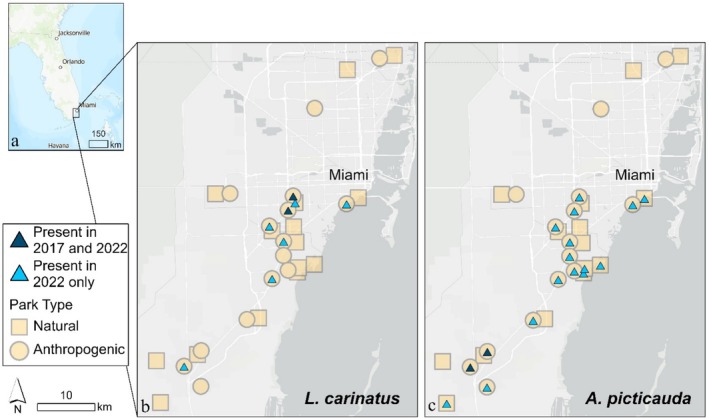
Map showing the study sites with (a) the location of the study area in Miami, Florida, USA and (b) and (c) the study area of 30 parks comprising both natural (squares) and anthropogenic (circles) parks in Miami‐Dade County. Occurrences of both (b) *L. carinatus* and (c) *A. picticauda* are shown with overlayed blue triangles, with light blue indicating sites with new presences in 2022. Both *A. picticauda* and *L. carinatus* were indicator species of the 2022 survey year (*p* < 0.03), and both were also indicator species for anthropogenic parks in 2022 (*p* < 0.01). Coordinate System: WGS1984. Map Sources: Esri, TomTom, Garmin, FAO, NOAA, USGS, OpenStreetMap.

Survey sites replicated those from Clements et al. (2019) and included 15 urban green spaces such as recreational and manicured areas (hereafter described as anthropogenic parks) and 15 parks where the natural habitat had been preserved (i.e., pine rockland, tropical hardwood hammock, and/or mangrove) across Miami‐Dade County (Figure 1). Natural and anthropogenic parks were paired geographically and spanned the same range of fragment sizes from 0.6 to 229 ha. None of the parks were contiguous with other parks or preserves.

### 2.2 Survey Methods

We conducted diurnal visual encounter surveys from March to May 2022 (peak local reptile and amphibian activity season), replicating the active survey methods performed 5 years earlier in 2017 (Clements et al. 2019). Visual encounter surveys (Enge, Robson, et al. 2004) were performed between 8 am and 7 pm during favorable weather conditions (e.g., minimal rain and temperatures above 18°C). This is the same set of weather conditions used to select 2017 survey dates, and thus while there was some variation in weather conditions between surveys within each year, mean conditions in 2017 and 2022 should be comparable. The time spent surveying each park replicated that of the 2017 surveys such that larger parks were sampled to the same depth on the species abundance curve as smaller parks (Clements et al. 2019). While each park was only surveyed once, this required that some of the larger parks were visited on multiple days to cover the entire area. Some parks had both natural and anthropogenic areas, but only natural areas were surveyed in parks that had been designated as natural parks and natural areas were not surveyed in parks that had been designated as anthropogenic parks, although we did survey ecotonal zones between the two habitat types. These choices mimicked those made in the 2017 surveys (Clements et al. 2019) because the goal of the 2022 surveys was to detect changes in the herpetofaunal community over the intervening 5‐year period. The habitats within the parks did not notably change over the course of the 5‐year period. Additional information about the study species encountered during these surveys can be found in the comprehensive overview of Florida reptiles and amphibians provided by Krysko et al. (2019).

Altogether, the surveys involved 384 person‐hours conducted by a team of 13 surveyors trained in the identification of local reptile and amphibian species. Survey hours were not divided evenly by the 13 surveyors (minimum 3 h, maximum 114 h), as they were dependent on availability. More than 80% of all survey hours were completed by those listed as authors on this manuscript, and the lead author was present at all sites to ensure consistent survey techniques were followed both within the 2022 surveys and with regard to the 2017 surveys. We found animals by sound and sight, scanning trees, tall grasses, bushes, anthropogenic structures within the parks, and other perching surfaces. We surveyed water bodies visually, paying particular attention to the shoreline. When necessary, we used binoculars to identify individuals out in water bodies or up in trees. We also searched under cover objects such as palm fronds, rocks, and anthropogenic materials (e.g., trash cans). When multiple natural habitat types (e.g., mangroves and pine rockland) were present in a park that had been designated as natural, all natural habitat types were thoroughly examined. Animals were only captured if necessary for identification and were then immediately released. Handling of animals was approved under University of Miami IACUC protocol 22‐016 in accordance with the ASIH/HL/SSAR Guidelines for the use of live amphibians and reptiles in field research.

### 2.3 Statistical Analyses

As in Clements et al. (2019), we used a second‐order jackknife to calculate estimated species richness in each park and the percentage of the estimated species that we observed (Gotelli and Colwell 2011). To ensure that our sampling in 2022 was to the same depth on the species abundance curve as in the 2017 surveys, we used a paired *t*‐test to determine if there was a difference in the percentage of estimated species that we observed between years, which could indicate a change in detectability.

We then investigated whether there was a difference in herpetofaunal abundance or richness based on survey year or habitat type (natural/anthropogenic) or their interaction. To address this question, we conducted factorial analyses of variance (ANOVAs) with year, habitat type (natural or anthropogenic), and their interaction as fixed effects and with park as a random effect to compare: (1) overall abundance and richness, (2) native abundance and richness, (3) non‐native abundance and richness, and (4) proportion of native individuals. Abundances and native richness were log‐transformed and the proportion of native individuals was arcsine‐transformed for normality. These analyses were conducted in JMP 17 (JMP Pro, Version 17.0.0 2022) and are available in the Appendix S1. We tested the residuals of all these analyses for spatial autocorrelation in R Version 4.0.2 using the *lctools* package with the number of nearest neighbors set to five (Ghosh 2006; Kalogirou 2017; R Core Team 2022) and made necessary adjustments to *p*‐values (Bivand et al. 2013; details in Appendix S1).

We then used ANCOVA to allow for inclusion of non‐native richness or abundance from 2017 as a covariate to account for pre‐existing differences in non‐native species across parks when analyzing the influence of environmental factors in 2022. Environmental factors that we considered included area of the park, native species richness (or abundance), habitat type (natural or anthropogenic), and connectivity. Connectivity was calculated using a patch‐based weighted sum (Winfree et al. 2005) that considers the distance that each park is from other parks of varying sizes. This was the same method used by Clements et al. (2019), who also investigated multiple dispersal constants, verifying that they caused no difference in the outcome of the analyses. We therefore chose to use a single dispersal constant (0.1) in the current study. Area, connectivity, and abundances were log‐transformed for normality. We evaluated all possible combinations of the environmental factors (omitting interactions) and selected the model with the lowest AICc using JMP 17. No models showed evidence of spatial autocorrelation (*p* > 0.3), which was tested in R Version 4.0.2 using the *lctools* package with the number of nearest neighbors set to five (Ghosh 2006; Kalogirou 2017; R Core Team 2022).

We also investigated whether the temporal change (2017 vs. 2022) in either abundance or richness was correlated between native and non‐native species using Pearson's correlations (data were normally distributed). This should illuminate whether there is a net negative effect of non‐native species accumulation on native herpetofauna in the 5‐year period of this study. We followed this by looking at some correlations between individual species that were either close relatives or particularly common species. All of the correlations between temporal changes in individual species abundances were tested using Spearman's correlations for non‐parametric data. We looked at the following species pairs: (1) *Anolis sagrei* and *A. carolinensis* , the most common non‐native and native species, respectively, (2) *A. carolinensis* and *A. cristatellus* , (3) *A. sagrei* and *A. cristatellus* , (4) *A. sagrei* and *Agama picticauda*, (5) *A. sagrei* and *Leiocephalus carinatus* , (6) *A. carolinensis* and *A. picticauda*, (7) *A. carolinensis* and *L. carinatus* , and (8) *A. picticauda* and *L. carinatus* . The first three comparisons look at competition between native/non‐native pairs of arboreal lizards with similar niches (Edwards and Lailvaux 2012), the next four comparisons look at the most common native and non‐native lizards and the most rapidly spreading predatory lizard species (Schoener et al. 2017), and the last comparison is between these rapidly spreading predators. All these analyses were conducted in JMP 17.

We wanted to ascertain if there was a difference in community composition between natural and anthropogenic habitats, as there was in Clements et al. (2019), and whether the indicator species for natural and anthropogenic habitats remained the same as 5 years before. We conducted a PERMANOVA using the Bray–Curtis distance metric (Anderson 2001; Clements et al. 2019) to determine if there was a difference in community composition between natural and anthropogenic parks using the new 2022 dataset. Any species found at a single park were excluded from the analysis, except for multiple species with low numbers that could be combined into a monophyletic group, such as *Hemidactylus* spp. The purpose of the community analysis is to determine which sites are more similar to each other and so a species found at only one site does not contribute to this analysis (Peck 2016). We relativized each row of the community matrix by person‐hours of survey time and each column by its total abundance. Relativizing by column was used to upweight rare species, particularly due to the overwhelming abundance of *Anolis* spp. found in the surveys. We followed this PERMANOVA with an indicator species analysis to determine which species were associated with natural versus anthropogenic parks (De Caceres and Legendre 2009).

We next determined whether there was a difference in community composition between survey years, again using a PERMANOVA. For this PERMANOVA, we combined our 2017 and 2022 data sets and then removed any species that were found in only one park‐year combination and could not be combined into a monophyletic group (i.e., *Hemidactylus* spp., *Nerodia* spp., *Plestiodon* spp., and *Pseudemys* spp.). Similarly, we corrected taxonomic names that differed between years and combined columns accordingly. For example, we had labeled blindsnakes *Rhamphotyphlops braminus* in 2017, but the currently accepted nomenclature is *Indotyphlops braminus* . We also misidentified *A. picticauda* as *Agama agama* in 2017, so we corrected this column from the 2017 data (Nuñez et al. 2016). We did not relativize this combined matrix by row or column because we were interested in comparing the two survey years and had used consistent survey effort to collect the data in both years. With this combined matrix, we ran a PERMANOVA with the Bray–Curtis distance metric and blocking by park. We followed this with an indicator species analysis to determine which species were most associated with 2017 versus 2022. All PERMANOVA and indicator species analyses were conducted in R Version 4.0.2.

Lastly, we were interested in whether we could identify predictors of abundance change for the two species that have proliferated most rapidly over the last 5 years: *A. picticauda* and *L. carinatus* . We again used ANCOVA model selection, with the same environmental factors described above, to evaluate whether habitat type (natural or anthropogenic), connectivity, or park area helped to predict the 2022 abundance of these species, with the covariate of their abundance in 2017. Abundance of *A. picticauda*, connectivity, and area were log‐transformed, while abundance of *L. carinatus* was square‐root transformed, and analysis was conducted in JMP 17. We also evaluated whether there was a spatial pattern to the increase in these species by looking for spatial autocorrelation, which was tested in R Version 4.0.2 using the *lctools* package with the number of nearest neighbors set to five (Ghosh 2006; Kalogirou 2017). The scripts and workflows for all analyses can be found in the Appendix S1.

## 3 Results

In total, we recorded 9535 individuals across 36 species in 2022. This represents an increase in individuals of 30% and in species of 14% over Clements et al. (2019). Only 7.7% of the individuals in the 2022 dataset were native, compared to 9.4% in Clements et al. (2019). Of the species recorded, 47% were native and 53% were non‐native. Based on the second‐order jackknife, there was no difference in the proportion of species observed between the 2017 and 2022 surveys (*p* = 0.14).

**FIGURE 2:**
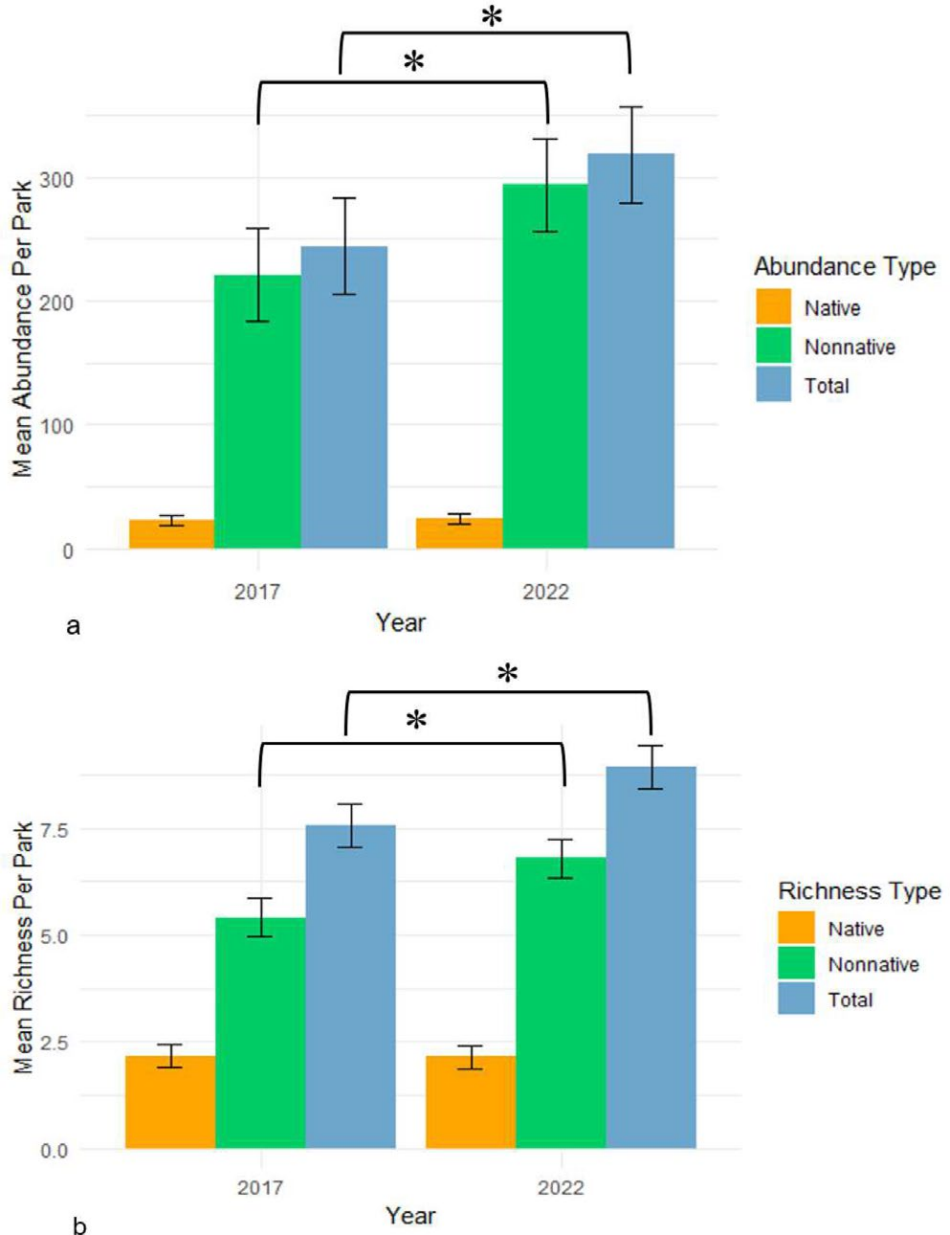
Bar graphs showing the difference in mean (a) abundance and (b) richness (±95% Confidence intervals) by year. Total abundance (*p* = 0.043) and richness (*p* = 0.001), as well as non‐native abundance (*p* = 0.032) and richness (*p* = 0.0012), increased significantly from 2017 to 2022, whereas native abundance and richness did not (*p* > 0.4).

Both overall abundance (*p* = 0.043) and richness (*p* = 0.001) of herpetofauna increased significantly in the 5 years between surveys. However, when separating native and non‐native species, we see this increase is only true for non‐native species, which increased 19% in richness (*p* = 0.0012) and 33% in abundance (*p* = 0.032). Native species richness and abundance did not notably differ from the 2017 surveys (*p* > 0.4; Figure 2). There was a marginally significant interaction between habitat type and year on non‐native richness (*p* = 0.058) in the direction of non‐native species increasing more rapidly in anthropogenic parks, but otherwise habitat type did not have a significant impact on richness, abundance, or proportion of native individuals (*p* > 0.09).

**FIGURE 3:**
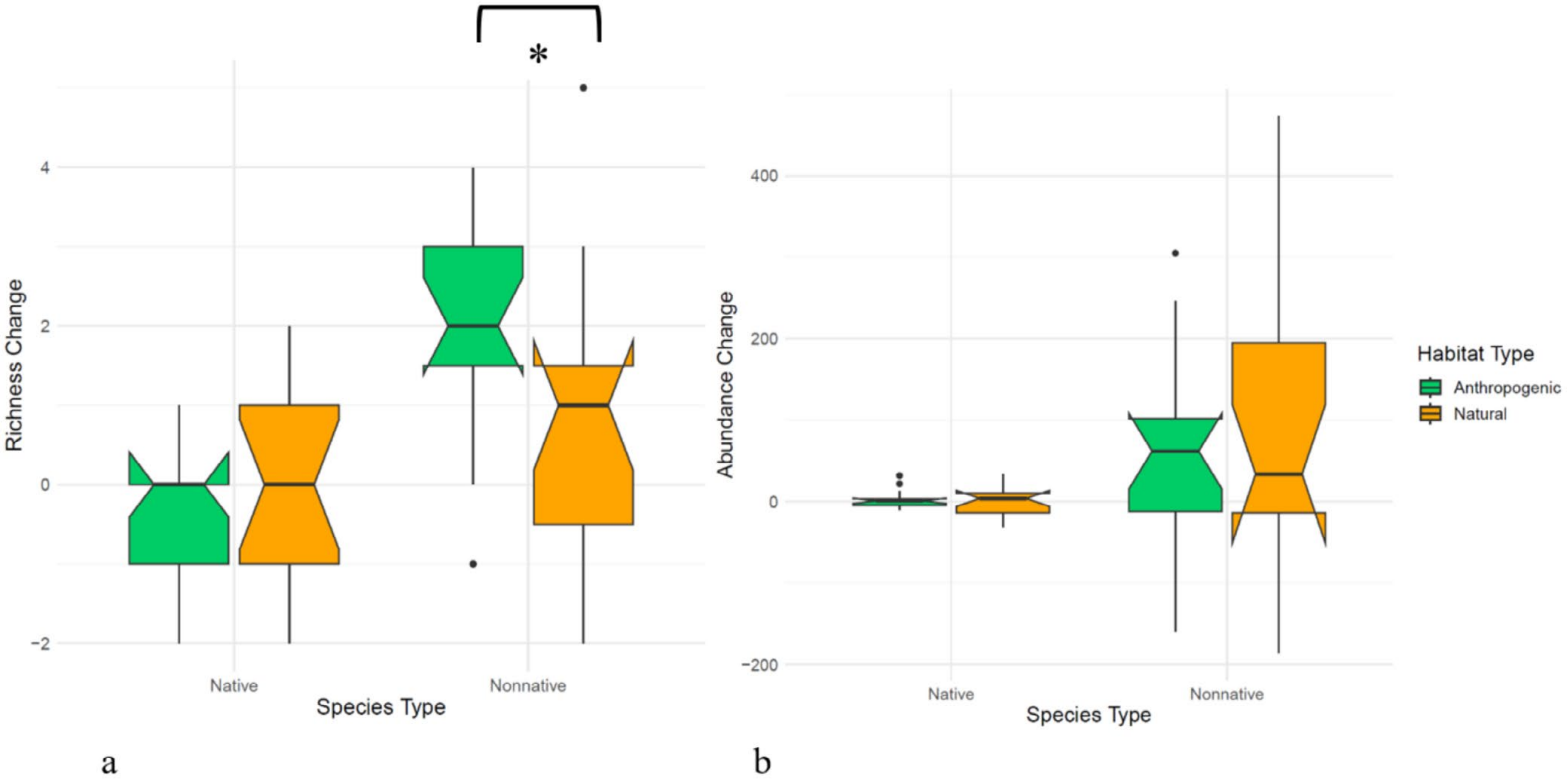
Boxplots comparing (a) change in richness and (b) change in abundance across years for natural (orange) and anthropogenic (green) parks across native and non‐native species. Boxplots represent the first and third quartiles, with the center line representing the median. Notches represent the 95% confidence interval around the medians. Whiskers are the minimum and maximum values, and dots represent outliers. Non‐native richness increased faster in anthropogenic than natural parks from 2017 to 2022 (*p* = 0.009). Native species richness did not change from 2017 to 2022, nor show any influence of habitat type. Increases in abundance were also not significantly related to habitat type for either native or non‐native species.

However, ANCOVA revealed that 2022 non‐native richness was higher in anthropogenic parks (estimate = −0.84, *p* = 0.009; Figure 3a), and in parks with greater 2022 native richness (estimate = 0.59, *p* = 0.03), as well as where there was a higher non‐native richness in 2017 (estimate = 0.59, *p* = 0.002) (Best model AICc = 118, *p* < 0.0001, *R* ^2^ = 0.68). Non‐native abundance in 2022 was best predicted by 2017 non‐native abundance (estimate = 0.44, *p* = 0.0073) and 2022 native abundance (estimate = 0.49, *p* = 0.0019), but not habitat type (Best model AICc = 50, *p* < 0.0001, *R* ^2^ = 0.80; Figure 3b). Park size and connectivity were not selected as important predictors for non‐native richness or abundance.

### 3.1 Species Analyses

As was true in 2017, the majority of the individuals recorded were from the genus *Anolis* (82% in 2022 and 86% in 2017). Non‐native species from the genus *Anolis* accounted for 75% of total individuals recorded. The most abundant herpetofauna species were *Anolis sagrei* (43.8%), *Anolis cristatellus* (18.0%), and *Anolis distichus* (12.3%). The most abundant native species were *Anolis carolinensis* (6.7%), *Sphaerodactylus notatus* (0.4%), and *Coluber constrictor* (0.3%). The most widespread native species were *A. carolinensis* (97% of parks surveyed) and *C. constrictor* (43%), and the most widespread non‐native species were *A. sagrei* (97%), *A. distichus* (87%), *Hemidactylus* spp. (70%), *A. picticauda* (57%), and *A. equestris* (53%). Five species, *A. cristatellus* , *A. picticauda*, *E. planirostris* , *I. iguana* , and *L. carinatus* , were found in ≥ 5 more parks in 2022 than in 2017. Two species, *Pseudemys nelsoni* and *Rhinella marina* , were found in ≥ 5 fewer parks in 2022 than they were in 2017. Of the 15 species found in at least five parks, only two were native.

**FIGURE 4:**
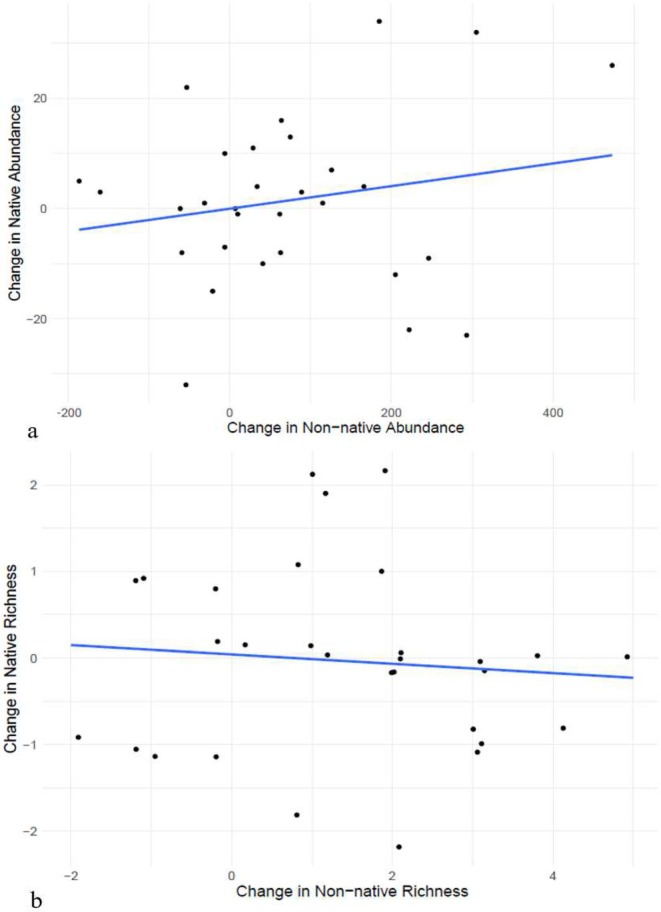
The change in non‐native species (a) abundance (*R* ^2^ = 0.04; *p* = 0.31) and (b) richness (*R* ^2^ = 0.008; *p* = 0.65) from 2017 to 2022 was not correlated with the change in native species abundance or richness, indicating that the increase in non‐natives is not having a detectable effect on natives. Each dot represents one park.

There was no correlation between the change in abundance of native and non‐native species (*p* = 0.31, *R* ^2^ = 0.04), nor between the change in richness of native and non‐native species (*p* = 0.65; *R* ^2^ = 0.008), indicating that the increase in richness and abundance of non‐natives is not having a detectable impact on the richness or abundance of natives (Figure 4). When we broke this down by species, there was also no correlation between the change in abundance for any of the species pairs tested (*p* > 0.10), with the exception of *A. picticauda* and *L. carinatus* , which increased together (*p* = 0.016; Spearman's *ρ* = 0.43).

Our 2022 PERMANOVA revealed a significant difference in community composition between natural and anthropogenic parks (*p* = 0.001), as was also true in the 2017 surveys (*p* = 0.001; Clements et al. 2019). However, our indicator species differed from those found in the prior survey (Clements et al. 2019). Our 2022 data revealed that a native snake, *C. constrictor* , was the indicator species for natural parks (*p* = 0.03), whereas in 2017, the indicator species of natural parks was the non‐native snake *I. braminus* (Clements et al. 2019). In 2022, we also found four indicator species for anthropogenic parks: *A. equestris* (*p* = 0.0001), *A. picticuada* (*p* = 0.0002), *A. distichus* (*p* = 0.002), and *L. carinatus* (*p* = 0.006). In 2017, *A. sagrei* and *A. equestris* were indicator species of anthropogenic parks (Clements et al. 2019).

**FIGURE 5:**
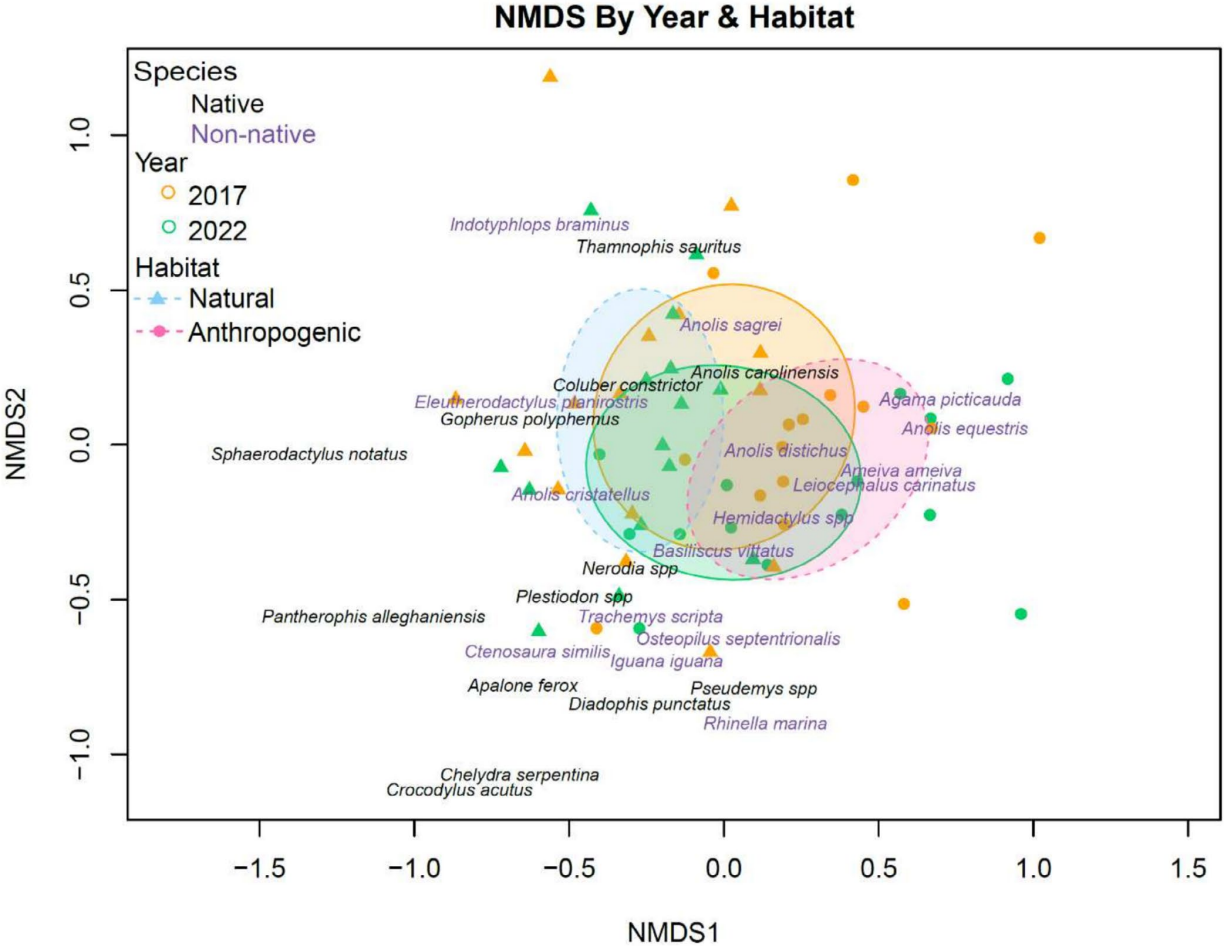
Non‐metric multidimensional scaling plot of herpetofaunal community composition (*k* = 3, stress = 0.173). There was a significant shift in community composition through time (2017 vs. 2022; *p* = 0.002). Community composition also differed significantly between habitat types (natural vs. anthropogenic) in both years (*p* = 0.001 in 2022 and *p* = 0.001 in 2017). Each park is represented twice, with 2017 surveys in orange and 2022 surveys in green. Circles represent anthropogenic parks, while triangles represent natural parks. The orange and green ellipses are 95% confidence intervals for 2017 and 2022, respectively, while the blue and pink ellipses are confidence intervals for natural and anthropogenic parks, respectively. The non‐native species are in purple and the native species are in black. The indicator species of 2022 were *A. picticauda* and *L. carinatus* , which were also indicator species of anthropogenic parks in 2022. Other indicator species of anthropogenic parks in 2022 included *A. distichus* and *A. equestris* , which was the only species that also was an indicator species of anthropogenic parks in 2017. The indicator species of natural habitat in 2022 was *C. constrictor* .

There was also a significant change in community composition through time (*p* = 0.002; Figure 5). The indicator species for 2022 were *A. picticauda* (*p* = 0.0001) and *L. carinatus* (*p* = 0.028), while there were no indicator species for 2017. Because *A. picticauda* and *L. carinatus* were revealed as the indicator species for 2022, and also were indicator species of anthropogenic parks in 2022, but not 2017, we wanted to further investigate the increase in these two species. While we only observed 10 *A. picticauda* in 2017 (Clements et al. 2019), we found 358 in 2022 (a 36‐fold increase). Even more notably, *A. picticauda* were found in only 2 parks in 2017 and in 17 parks in 2022 (Figure 1c). In 2017, *L. carinatus* was found in just 2 parks and only 4 individuals were observed, whereas in 2022, we found *L. carinatus* in 8 parks (Figure 1b) and 87 individuals were observed (a 22‐fold increase). When blocking by park, the abundance of *A. picticauda* was significantly higher in 2022 (*p* = 0.02), while the abundance of *L. carinatus* was only marginally higher in 2022 (*p* = 0.07). Through model selection, we found that the 2022 *A. picticauda* abundance was higher in anthropogenic parks (estimate = 0.7, *p* = 0.004) and parks with a higher abundance of *A. picticauda* in 2017 (estimate = 1.05, *p* = 0.06; best model AICc = 103, *p* = 0.001). The 2022 *L. carinatus* abundance was marginally higher in anthropogenic parks (estimate = 0.43, *p* = 0.057) and parks with a higher abundance of *L. carinatus* in 2017 (estimate = 2.5, *p* = 0.0004; best model AICc = 115, *p* = 0.0001). There was a marginal geographic pattern of spatial autocorrelation between parks with the greatest increase in *A. picticauda* (*p* = 0.05), but no spatial autocorrelation for *L. carinatus* (*p* = 0.48). Anecdotally, the authors believe that there is a relationship between parks where agamas are increasing and the presence of cement or other hard man‐made structures either inside or along the edge of the park, which aligns with recent findings (Mothes and Searcy 2024).

## 4 Discussion

Our surveys indicate that the non‐native herpetofaunal community of Miami‐Dade County has experienced rapid increases in both abundance and richness within just 5 years. Between 2017 and 2022, we found that the richness of non‐native species increased across both natural and anthropogenic parks by an average of 1.4 species per park. This appears to be a true increase in richness rather than an increase in detectability, as the percentage of the predicted species pool observed did not change significantly between years. In addition, because the increase in species observed was attributed solely to the non‐natives, we believe this represents a real expansion in the non‐native community and is a considerable increase in non‐native richness in a relatively short period of time. We also found that the abundance of non‐native species increased across both natural and anthropogenic parks. While there are certainly differences in detectability across herpetofauna species based on behavior and activity, those differences should be consistent between 2017 and 2022, making our abundance comparisons across time valid. While it is feasible that the increased abundances detected in 2022 were a result of the surveyors being more skilled, there is no obvious reason why this would be true, and if this was the driving factor, we would expect increases in natives and non‐natives to be of similar magnitude. Compared to the 2017 surveys, non‐native abundance increased by 32.7% (a significant increase) while native abundance increased by only 6.5% (a non‐significant change). In the 2022 surveys, 92.3% of individuals were non‐native (compared to 90.6% in 2017; Clements et al. 2019). Even among the 7.7% of individuals that we recorded as native, there is an important caveat to consider. The most abundant native species recorded was the green anole ( *A. carolinensis* ), but studies have reported that the green anole population in Miami‐Dade County consists of morphologically similar hybrids between the native *A. carolinensis* and the non‐native Cuban green anole ( *Anolis porcatus* ; Wegener et al. 2019). If we were to reclassify *A. carolinensis* as non‐native due to this hybridization, then non‐natives would make up an astounding 99% of all individuals recorded. This staggering abundance of non‐native individuals shows why South Florida provides unique insights into the spread and effects of non‐native species (Fujisaki et al. 2009).

Over the last 5 years, non‐native species richness in this ecosystem has increased by 18% while non‐native abundance has increased by an even greater 33%. With both non‐native abundance and richness on the rise, we expected that we might see negative impacts on native species. We know that non‐natives can cause rapid decreases in native populations and even extinctions in a < 5‐year time period, as was the case with the non‐native brown tree snake in Guam (Savidge 1987; Rodda et al. 1997). However, at least at the community level, there is no clear signature of such an effect in Miami‐Dade County. Native abundance and richness remained relatively constant over the last 5 years at each of our study sites, regardless of increases in non‐native abundance and richness. While the lack of a non‐native effect on native species has certainly been documented in other systems (e.g., herbaceous plants; Davis et al. 2015), it is counter to the global trend. In general, native responses to invasion become more negative as both the abundance and trophic level of the invading species increase (Gleditsch and Carlo 2010; Bradley et al. 2019). It is therefore interesting that, despite a high abundance of invading species spanning many trophic levels, there is not a negative impact of invaders on the richness or abundance of native herpetofauna in South Florida.

It is important to note that measurements of richness and abundance look at detrimental effects on native species at the community level, but that individual non‐native species may still be affecting individual native species even where community level responses are not seen. For example, the invasion of cane toads ( *Rhinella marina* ) in northern Australia resulted in population declines in several species of native predatory lizards *Varanus panoptes* , *V. mertensi* , and *V. mitchelli* ), but also a population increase of one of *V. panoptes* 's native prey species, the lizard *Amphibolurus gilberti* (Doody et al. 2009). In this case, overall native abundance would not have reflected the severity of native predator species' declines due to the offsetting increase in the native prey species. We tested for non‐native effects at the individual species level, but we found no correlation between changes in abundance of any two individual species (other than a positive correlation between *A. picticauda* and *L. carinatus* , both non‐native species). However, many native species were only detected in these surveys at very low abundances or at very few locations, limiting replicability across sites and therefore our ability to detect significant correlations. We did investigate whether there was a correlation between the change in the most abundant non‐native, *Anolis sagrei* , and the most abundant native, *A. carolinensis* , but found no evidence of this. However, we only looked at changes in abundance, while other studies have demonstrated that *A. carolinensis* may be affected behaviorally by non‐native encroachment. The native species demonstrates a niche shift toward greater arboreality in the presence of *A. sagrei* (Edwards and Lailvaux 2012; Stuart et al. 2014). This behavioral and associated morphological (Glossip and Losos 1997; Stuart et al. 2014) shift demonstrates adaptation by a native species to avoid direct negative effects from the encroachment of a non‐native.

Some non‐natives are notorious for their destructive effects on native species, such as *Python bivittatus* and *Salvator merianae*. We detected both species at our survey sites for the first time in 2022, despite ongoing conservation efforts to contain and eradicate populations of these species. Significant declines in native mammals have been documented in Everglades National Park, particularly in areas with high *P. bivittatus* proliferation (Dorcas et al. 2012). Like *P. bivittatus* , *S. merianae* is a generalist predator, preying on American alligator ( *Alligator mississippiensis* ) and red‐bellied cooter *Pseudemys nelsoni* ) eggs (Mazzotti et al. 2015). In 2021, the Florida Fish and Wildlife Conservation Commission (FWC) introduced new regulations regarding the keeping, breeding, trading, and selling of 16 prohibited reptile species, including *P. bivittatus* and *S. merianae* (FLA 2021). However, these measures have not stopped the dispersal of individuals from well‐established populations. Furthermore, non‐native species can have more subtle impacts on native species, such as the spillover of non‐native parasites (e.g., spillover of a harmful Asian parasite ( *R. orientalis* ) from *P. bivittatus* to 14 native snake species; Miller et al. 2017; Miller et al. 2020). While beyond the scope of our study, it is important to recognize that non‐native impacts on native species extend beyond decreases in abundance or richness.

We also investigated potential correlations between changes in non‐native species. Previous studies have documented that ecologically analogous non‐native *Hemidactylus* spp. are unable to stably co‐exist, resulting in the competitively dominant *H. mabouia* rapidly displacing *H. garnotii* in their sympatric range in central and southern Florida (Meshaka Jr 2000; Short and Petren 2012). However, we did not see any evidence in our surveys of a negative correlation between the non‐native species pairs that we investigated. Again, this may be due to few co‐occurrences of certain species preventing the replicability needed to test this effect. For example, at Evelyn Greer Park, the number of *Ameiva ameiva* observed decreased dramatically from 2017 to 2022, while *A. picticauda* were detected there for the first time in 2022, and at a high abundance. While this could indicate a potential displacement of *A. ameiva* by *A. picticauda*, the two species do not co‐occur at enough sites to test this hypothesis. Similarly, on multiple occasions, we observed *A. picticauda* preying on non‐native *Anolis* species and pursuing the native *A. carolinensis* . As a large predatory lizard, *A. picticauda* could negatively impact populations of smaller species, although we could not detect such an effect.

We also investigated the impact of habitat type (natural or anthropogenic) on non‐native increases in richness and abundance. Clements et al. (2019) found no significant difference in native or non‐native herpetofaunal abundance or richness based on habitat type. While it remains true that there is no difference in the richness or abundance of native species between habitat types in 2022, we now see that, importantly, the increase in non‐native species is more rapid in anthropogenic areas. Disturbances to natural habitats have been shown to facilitate the diversity and abundance of non‐native plant species as well (Jauni et al. 2015). Given that there was no difference in non‐native penetration of natural vs. anthropogenic parks during the 2017 surveys, there must have been some previous time point in the herpetofauna invasion of South Florida when non‐native species were not inhibited by natural habitat. However, for the species currently expanding in Miami, it seems to be the case that the natural areas serve as an impediment to spread, demonstrating an important benefit of the preservation of natural parks, which may also serve as a reservoir for native species, similar to what has been seen in other systems (Chace and Walsh 2006). In our study, we saw that the indicator species of natural parks in 2022 was the native *Coluber constrictor* , demonstrating that these natural habitat areas may be a refuge for common native species. These findings highlight the importance of conserving natural areas to slow the spread of some non‐native species and to support native populations.

The primary indicator species for the change in community composition from 2017 to 2022 were *Agama picticauda* and *Leiocephalus carinatus* , non‐native species that have increased in abundance 36‐fold and 22‐fold, respectively, since 2017. These two species were also new indicator species for anthropogenic parks in 2022. Taken together, these results demonstrate that these two non‐native species have increased rapidly in distribution and abundance in anthropogenic areas across Miami‐Dade County in just the last 5 years. The current *A. picticauda* population is thought to have been introduced in southern Miami‐Dade in 1992 as a result of Hurricane Andrew (Enge, Krysko, et al. 2004), with DNA analyses indicating multiple subsequent introductions contributing to their current genetic diversity (Nuñez et al. 2016). *Leiocephalus carinatus* was first reported in Florida in Palm Beach County in 1958 and has subsequently spread toward Miami (Smith and Engeman 2004). Both *L. carinatus* and *A. picticauda* are predisposed to succeed in human‐disturbed environments, as in their native range both species prefer open habitats with rocky structures for basking (Neel et al. 2020; James and Porter 1979). In urban environments, impervious surfaces, such as parking lots, provide a suitable substitute for their preferred habitats (Meshaka Jr. et al. 2022). The abundance of *A. picticauda* can also be positively predicted by the presence of human structures such as dumpsters (Mitchell et al. 2021), which likely serve as refugia as well as attract prey. Anthropogenic parks contain plentiful warm cement basking locations including curbs, parking lots, and sidewalks, while also providing shade and protection from predators through shrubbery, trees, etc. (Moore and Smith 2006). New preliminary research in Miami‐Dade County suggests that species that prefer hotter perches, like *A. picticauda* and *L. carinatus* , are those increasing most rapidly in abundance, an interesting topic for further study (E. Afkhami Searcy, personal communication).

Both *A. picticauda* and *L. carinatus* are clearly dispersing rapidly, as *A. picticauda* and *L. carinatus* were both detected in only 6.6% of parks in 2017 yet were found in 57% and 27% of parks, respectively, in 2022. *Agama picticauda*, in particular, has been documented spreading rapidly within Florida and even beyond in the past few years, with new records recently recorded in 20 Florida counties and 5 additional states (Enge 2024). Both species likely use similar dispersal methods to other local non‐native lizards, especially the extremely widespread and abundant *A. sagrei* , for which vehicular rafting is a theorized dispersal method (Campbell 1996). Similarly, *A. picticauda* has also been documented using cars and interstates for dispersal (Moore 2019) and is speculated to hitchhike on freight using railways (Gray 2020). There is also evidence for dispersal of both *L. carinatus* and *A. picticauda* being aided by the nursery industry, as researchers have witnessed *L. carinatus* perched on landscaping vegetation piles (Smith and Engeman 2004) and 72% of the suspected origins of *A. picticauda* subpopulations are located within 0.5 km of plant nurseries, importers, or exporters (Gray 2020). These modes of dispersal help explain the increase in non‐native species in anthropogenic parks, as natural areas experience less landscaping and park‐goers, as well as fewer other hallmarks of urbanization that would facilitate the spread of these species. It is worth noting, however, that all natural parks in this study are within the urban matrix and even for the largest ones none of their interiors are more than 700 m from an urban area (Clements et al. 2019), which may play an important role in why we see such a high abundance of non‐natives even in our natural park sites.

Despite a long history of non‐native herpetofauna establishment in South Florida, non‐native richness and abundance have continued to increase over the last 5 years, while native richness and abundance have remained relatively constant. As such, there does not appear to be a negative impact of non‐natives on native richness or abundance over the 5‐year time frame in which this study was conducted. Further studies will be necessary to determine if this remains true or if native populations begin to decline more widely because of direct competition/predation from non‐natives beyond the losses that have already occurred due to loss of habitat. It is possible that the native populations experienced a significant decrease at some point in the past prior to the 5‐year window of our study. As discussed in Clements et al. (2019), there are some native species in Miami‐Dade County, such as skinks (*Plestiodon* spp.), that are now recorded at lower abundances and/or occurrences than surveys from the early 2000s seem to suggest (Enge, Robson, et al. 2004). Our study would not be able to determine if native species were already lost, or had reached a new, but lower, stable population size prior to the 2017 surveys, as a result of habitat loss, non‐native species encroachment, or some other factor. Additionally, there is the possibility of a prolonged lag in non‐native impacts, which could be attributed to novel changes in the non‐native species in the form of prey switching or evolution in the invaded range to become more competitive (Crooks 2005). This could occur over decades, as seen with the introduction of the non‐native snail *Batillaria attramentaria* off the California coast from 1932 to 1955, which was reported to co‐exist with the native snail *Cerithidea californica* for several decades, until a sustained decline of the native was recorded in 1999 (Byers 1999). Such a prolonged lag would not be detected in our study as several of the non‐native introductions have occurred relatively recently. Our study also cannot assess whether there has been any change in native or non‐native abundance/richness in larger, contiguous natural habitat areas since our focus was on urban Miami. Future studies should investigate these dynamics in larger natural areas such as the Everglades, where the abundance of non‐native species remains lower than in the urban core (~16% non‐natives in Loxahatchee National Wildlife Refuge; Howell et al. 2021).

Native abundance and richness did not differ significantly between natural and anthropogenic parks over the 5 years, but anthropogenic parks demonstrated the highest increase in non‐native richness. This finding suggests that prioritizing the conservation of natural areas will be critical when attempting to inhibit the spread of non‐native species. While non‐native herpetofauna individuals already comprise > 90% of the community in Miami‐Dade County, our study demonstrates that both richness and abundance of non‐native herpetofauna continue to increase. These increases will likely continue as the newer non‐natives spread to additional localities and increase in abundance, urban development continues to disrupt natural habitats, and new species continue to be introduced. Continued monitoring of the world's most heavily invaded herpetofauna community will be critical for understanding potential impacts of non‐natives on native species, as well as the factors that influence the spread of non‐native species.

## Author Contributions

**Stephanie L. Clements:** conceptualization (equal), data curation (equal), formal analysis (equal), investigation (equal), methodology (equal), visualization (equal), writing – original draft (equal), writing – review and editing (equal). **Frederick M. Ackerman:** data curation (equal), formal analysis (equal), investigation (equal), writing – original draft (equal), writing – review and editing (equal). **Isabella M. Olensky:** investigation (equal), writing – original draft (equal), writing – review and editing (equal). **Elizabeth C. White:** investigation (equal), writing – original draft (equal), writing – review and editing (equal). **Millie E. Rogers:** investigation (equal), writing – original draft (equal), writing – review and editing (equal). **Christopher A. Searcy:** conceptualization (equal), funding acquisition (equal), investigation (equal), methodology (equal), writing – original draft (equal), writing – review and editing (equal).

## Disclosure

Research involving animals: Procedures involving animals were approved under IACUC protocol number 22‐016 at the University of Miami in accordance with the ASIH/HL/SSAR Guidelines for use of live amphibians and reptiles in field research.

Permits: This work was conducted under a permit from Florida Fish and Wildlife Conservation Commission (#LSSC‐16‐0013C) and under a permit from Miami‐Dade County Parks and Recreation Department (#345).

## Conflicts of Interest

The authors declare no conflicts of interest.

## Supporting information


Appendix S1

## Data Availability

The data, workflows, and scripts that support the findings of this study are available in the Supporting Information of this article.
